# First Worldwide Report of a Total Colectomy with the Hugo RAS Platform

**DOI:** 10.3390/jcm13206071

**Published:** 2024-10-11

**Authors:** Marisa Domingues dos Santos, Pedro Brandão

**Affiliations:** 1 Colorectal Surgery Unit, Unidade Local de Saúde de Santo António, Centro Hospitalar Universitário de Santo António, 4050-342 Porto, Portugal; pedronunobrandao.cirurgia1@chporto.min-saude.pt; 2 UMIB—Unit for Multidisciplinary Research in Biomedicine, ICBAS—School of Medicine and Biomedical Sciences, University of Porto, 4050-346 Porto, Portugal; 3 ITR—Laboratory for Integrative and Translational Research in Population Health, 4050-600 Porto, Portugal

**Keywords:** Hugo RAS total colectomy, robotic total colectomy, minimally invasive surgical procedure, robotic colorectal surgery, familial adenomatous polyposis, robotic technical surgery aspects

## Abstract

**Background**: Compared with the da Vinci platform, there is limited experience with the Hugo RAS^®^ platform for colorectal surgery in Europe. This difference is especially notable when considering complex procedures such as total colectomy. **Aim**: To demonstrate the feasibility and safety of using the Hugo RAS^®^ (Medtronic, Minneapolis, MN, USA) platform for total colectomy. Clinical case: An 18-year-old female patient with Familial Adenomatous Polyposis (FAP) and a BMI of 19 underwent a total colectomy with ileorectal anastomosis using the Hugo RAS^®^ platform. The procedure lasted 253 min without complications. The postoperative period was uneventful, and she was discharged from the hospital on the third postoperative day. **Conclusion**: The Hugo RAS^®^ platform is an emerging minimally invasive robotic that can be used even for total colectomy with proper patient selection. The placement and choice of arms and trocars were crucial to obtaining a similar operative time to the standard laparoscopic approach. The certification of Hugo’s new instruments, such as energy devices and staplers, will make this platform even more competitive.

## 1. Introduction

The worldwide implementation of robotic surgery for colorectal procedures has been progressing gradually but slowly due to economic aspects. It is undeniable that robotic platforms enhance three-dimensional visualization and improve dexterity, providing better visualization of pelvic structures, improved dissection, and greater ease in creating anastomoses. Moreover, they are more ergonomic for the surgeon. These advantages can translate into clinical practice by reducing morbidity, shortening the length of hospital stay, and providing better long-term results than the laparoscopic approach [1]. The drawbacks include extended operative time, higher initial investment, and increased consumable costs [1].

The da Vinci^®^ platform by Intuitive Surgical, Sunnyvale, CA, USA, has been installed in reference centers for over two decades, and continuous upgrades have made it the robotic system of reference. Emerging robotic platforms aim to offer alternatives to this pioneer system. For this to happen, new systems must be competitive in terms of feasibility, safety, outcomes, and associated lower costs than the da Vinci robotic-assisted system.

The advantages of robotic surgery are well-established for segmental colon resection [1,2]. However, the same certainty is absent when focusing on total colectomy [3]. Total colectomy is a more complex procedure that requires working in all four quadrants. Moving the patient-side surgical cart during surgery adds complexity and is associated with longer operative times. The new model of da Vinci**^®^**, the Xi boom-mounted system, partially resolves this problem [4]. Even so, the number of total colectomies performed worldwide with a robotic system remains limited [5].

The Hugo RAS**^®^** is one of the emerging and promising robotic systems. This modular platform allows surgery using up to four arm carts [6,7]. It has been available in our center for one year and a half. To date, we have performed eighty colorectal procedures assisted by the Hugo RAS**^®^** system.

We describe the first total colectomy performed with the Hugo RAS**^®^**, which was published worldwide. We highlight the technical aspects of the procedure to ensure a safe and effective outcome.

## 2. Materials and Methods

### 2.1. Patient

**Figure 1:**
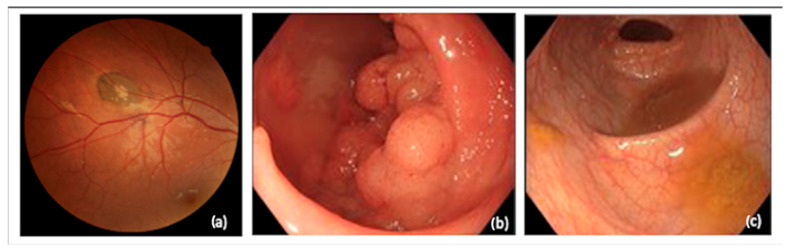
Clinical aspects: (**a**) CHRPE in right eye ocular fundus (**b**) Hundreds of adenomatous polyps and laterally spreading lesions throughout the colon (**c**) There was a small number of polyps in her rectum that were removed by endoscopy.

The selected patient is an 18-year-old female with an index case of Familial Adenomatous Polyposis (FAP). The diagnosis was suspected after an ophthalmologic exam revealed the presence of congenital hypertrophy of the retinal pigment epithelium (CHRPE) in both eyes (Figure 1a).

Based on this unexpected finding, the patient underwent a colonoscopy (Figure 1b) and a genetic study that confirmed the diagnosis. She had a heterozygous pathogenic variant in the APC gene. Her parents’ colonoscopies and genetic studies were normal.

She was psychologically stable and capable of following medical advice. She and her family have regular medical appointments at the hereditary cancer clinic.

She was referred for colorectal surgical consultation because she had more than 500 polyps, some of which were laterally spreading lesions with low- and high-grade adenomatous changes. She had a small number of polyps in her rectum removed by endoscopy (Figure 1c).

After discussing the pros and cons of a total colectomy with ileorectal anastomosis versus a reconstructive proctocolectomy, it was decided to perform a total colectomy with ileorectal anastomosis and close endoscopic surveillance of the rectum postoperatively.

The surgery was scheduled for April 2024. The patient was hospitalized and had completed surgical intestinal preparation with oral antibiotics and laxatives.

### 2.2. Platform Hugo RAS

A Hugo RAS^®^ platform was utilized for robotic surgery. Four arms and one extra tower with a monitor were used. The console was placed in the operating room. The robotic instruments included the camera, monopolar curved shears, bipolar Cadiere forceps, and Cadiere forceps. The instruments for the assistant port included a Medtronic stapler (Signia™ (Minneapolis, MN, USA) stapling system), Covidien EEA circular stapler, a device for insufflation and smoke evacuation and filtration, laparoscopic shears, Hem-o-lok endoscopic applicator, and Hem-o-lok clips. An advanced energy device was not used.

The arms of the Hugo RAS^®^ were placed on each side of the patient.

M Medtronic technical support was present during the surgery.

### 2.3. Surgical Procedure

#### 2.3.1. Summary

The surgery was performed sequentially on the right colon (Phase II) and the left colon and pelvis (Phase IV). The division of the rectum was performed with a stapler, marking the end of the robotic phase and the start of the laparoscopic phase (Phase V).

#### 2.3.2. Description of the Procedure Steps

Phase I—Initial steps and System Configuration

A—Port placement principles

**Figure 2:**
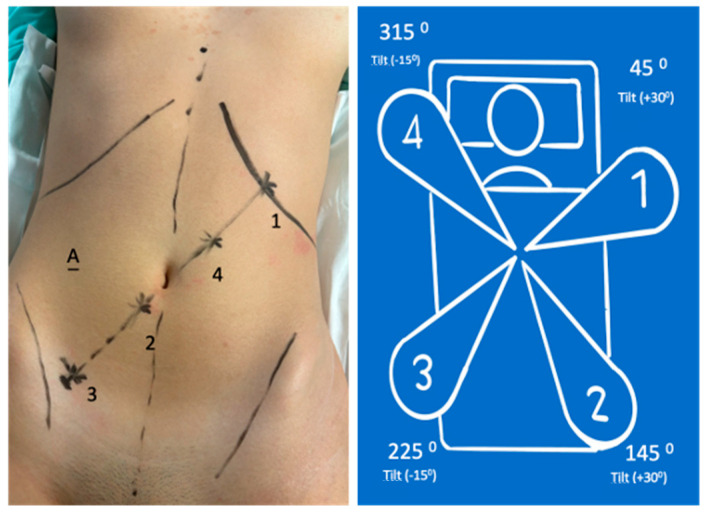
Port positions and system configuration for right colectomy.

The patient was positioned on the operating table at a height of 60 cm. The schematic port placement was marked on the patient (Figure 2).

Four ports were positioned diagonally from the left hypochondrium to the right iliac fossa. The robotic trocars included two measuring 8 mm and two measuring 11 mm. Of the 11 mm robotic trocars, one was used for the camera; the other was utilized with traditional laparoscopic instruments when needed, thus avoiding the need for a second assistant trocar on the patient’s left side. A 12 mm trocar was placed on the lateral edge of the transition from the upper right quadrant to the lower right quadrant. This trocar was used for insufflation, smoke evacuation, filtration system (Palliare™), Hem-o-lok application, Signia™ stapler utilization, etc.

B—Patient and bed position

A laparoscopic approach was used to position the patient and prepare the anatomical field for starting robotic colectomy. The table was placed at 10 degrees Trendelenburg and 10 degrees left lateral tilt.

C—System Configuration

Docking the arms: (Docking time: 3:25 min)

1. 8 mm (arm in lateral-superior left position): Tilt (+30°)—dock—45°—reserve;
2. 11 mm (arm in the lateral-inferior right position): Tilt (+30°)—dock—145°—endoscope/assistant port;
3. 8 mm (arm in the lateral-inferior right position): Tilt (−15°)—dock—225°—surgeon’s left hand (bipolar);
4. 11 mm (arm in lateral-superior left position): Tilt (−15°)—dock—315°—surgeon’s right hand (monopolar curved shears); also used for laparoscopic Hem-o-lok application.

Laparoscopic port:

- Assistant port and insufflation/aspiration system

Phase II—Assisted robotic right colectomy

**Figure 3:**
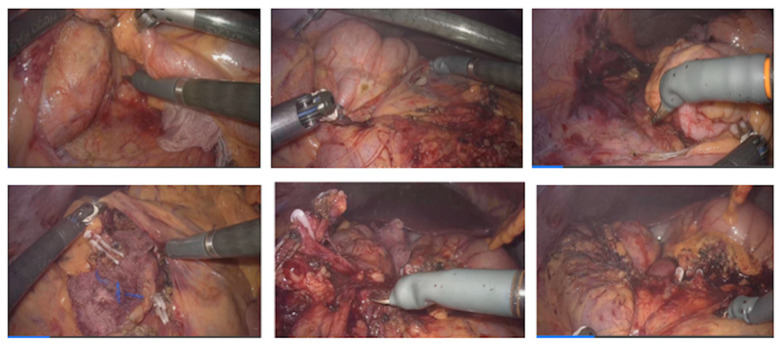
Aspects of robotic right colectomy surgical steps.

The steps of the robotic right colectomy are shown in Figure 3.

Phase III—System Reconfiguration (Docking time: 2:55 min)

**Figure 4:**
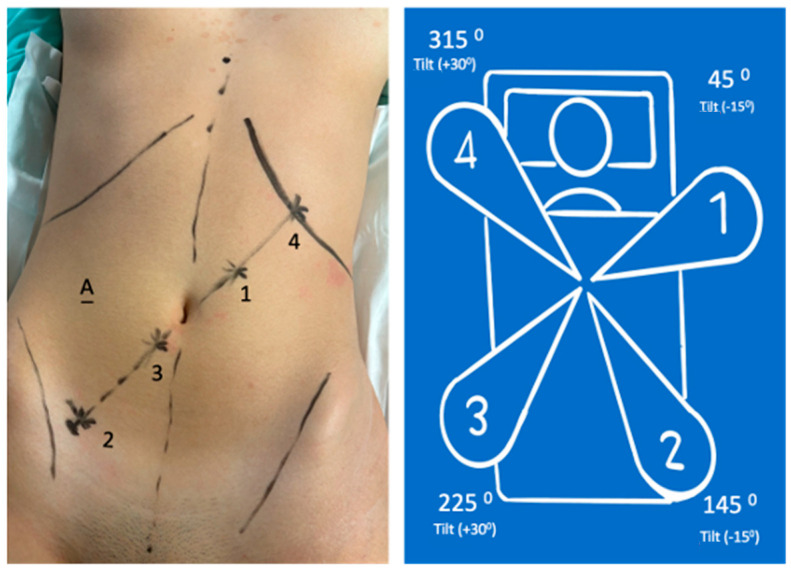
Port position (the same) and System reconfiguration for left colectomy.

The patient’s position was changed to the same Trendelenburg (10 to 15°) with a right lateral tilt of 10 to 15°. Arms were re-docked (Figure 4):

1. 8 mm (arm in the lateral-superior left position): Tilt (−15°)—dock—45°—surgeon’s left hand with Cadiere grasper;
2. 8 mm (arm in the lateral-inferior right position): Tilt (−15°)—dock—145°—surgeon’s right hand (monopolar curved shears);
3. 11 mm (arm in the lateral-inferior right position): Tilt (+30°)—dock—225°—endoscope;
4. 11 mm (arm in the lateral-superior right position): Tilt (+30°)—dock—315°—reserve (Cadiere bipolar forceps).

Laparoscopic port:

- Assistant port and insufflation/aspiration system

Phase IV—Assisted robotic left colectomy

**Figure 5:**
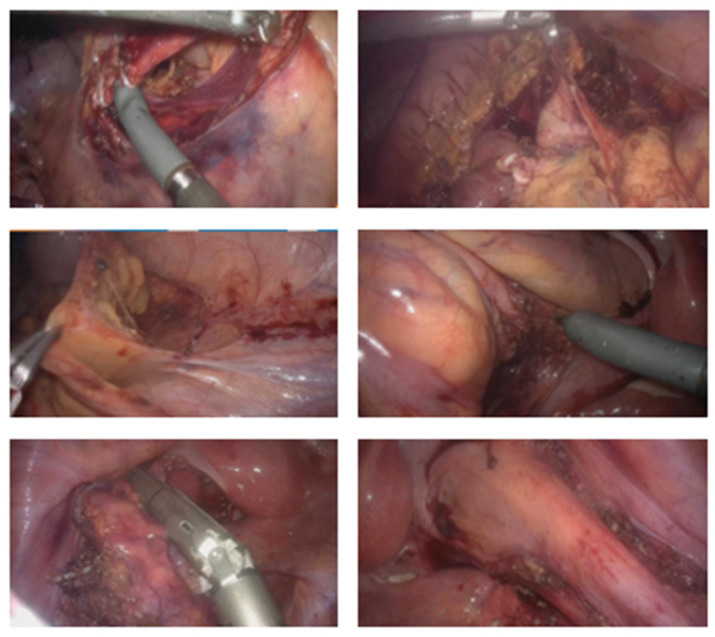
Aspects of robotic left colectomy surgical steps.

The steps of the robotic left colectomy are shown in Figure 5.

Phase V—Laparoscopic Assisted phase.

A. Suprapubic incision;
B. Extraction of the surgical specimen;
C. Ileorectal anastomosis.

#### 2.3.3. General Description of the Procedure

Phase I—Configuration of the System

For the robotic procedure, the patient was placed in a modified lithotomy position. After pneumoperitoneum was created with a Veress needle at the incision site for the camera, a total of five ports were placed, as shown in Figure 2.

The patient was then positioned in 10° of Trendelenburg and 15° of left lateral tilt.

The robotic arms were docked (Figure 2).

Phase II—Right Colectomy (Figure 3)

The procedure began on the right side with entry into the retroperitoneum in the avascular portion of the mesentery between the superior mesenteric vein and the ileocolic vessels. The retroperitoneal structures, including the third portion of the duodenum and the pancreas, were bluntly dissected posteriorly. The ileocolic vessels were isolated and divided. Dissection proceeded along the superior mesenteric vein axis to identify the right colic artery and vein (when present) and the middle colic artery and vein. All vascular pedicles were controlled with Hem-o-lok clips. The mesentery of the right colon was mobilized from medial to lateral, leaving behind the pancreas, duodenum, and retroperitoneal structures. The greater omentum was then opened to enter the lesser sac and complete the mobilization of the right colon.

Phase III—System Reconfiguration

The robotic arms were detached from the trocars, and the patient was repositioned to 15° of right lateral tilt. The robotic arms were reconnected to the trocars, and the instruments were changed, as shown in Figure 4.

Phase IV—Robotic Left Colectomy (Figure 5)

The inferior mesenteric artery and vein were divided, and a medial-to-lateral approach detached the left mesocolon from the pancreas tail, the ureter, and the gonadal vessels. The left paracolic gutter was opened to the splenic flexure, which was taken down. The great omentum was completely disconnected from the left side of the transverse colon, which was also completely released. After the sigmoid was detached from the lateral attachments, mesorectal partial dissection was performed, and the rectum was divided below the colorectal transition. All vascular pedicles were controlled with Hem-o-locks.

Phase V—Assisted Laparoscopic Phase

A Pfannenstiel incision was made, and the ileum was divided. After the division of the ileum and extraction of the surgical specimen, a 28 mm EEA circular stapler anvil was introduced into the ileum. The ileorectal anastomosis was performed after transanally introducing the body of the stapler.

The integrity of the ileorectal anastomosis was tested with methylene blue.

The ports were closed with Vicryl™ sutures and staples.

**Figure 6:**
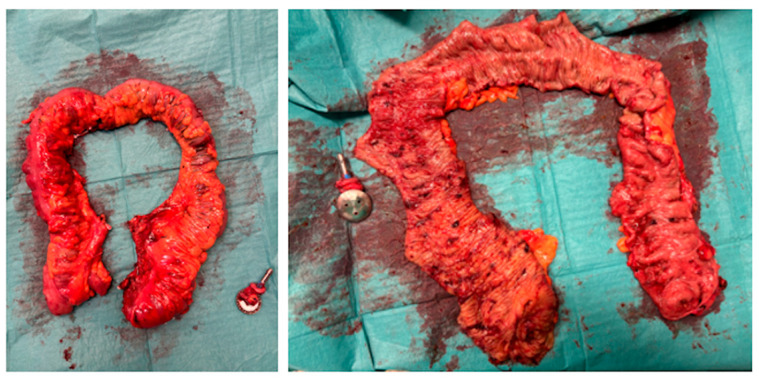
Surgical specimen (colon close and open).

Figure 6 shows the surgical specimen.

Table 1 shows the summary of the main technical aspects of the procedure.

## 3. Results

The operating time was 253 min. Blood loss was minimal.

The postoperative period was uneventful. She passed flatus and started enteral nutrition on the first postoperative day. She initiated a liquid diet on the second day. She passed stools and was discharged from the hospital on the third postoperative day.

The histopathological study revealed Macrocopy—an ileocecal colectomy piece comprising a 5 cm ileum segment, 8 cm ileocecal appendix, and 80 cm colon plus anastomosis rings. Microscopy—Colon with multiple adenomas of predominantly tubular architecture, some tubulovillous, most with low-grade dysplasia, but with several foci of high-grade dysplasia in a context of Familial Adenomatous Polyposis (without adenocarcinoma). Anastomosis rings without adenomas.

**Figure 7:**
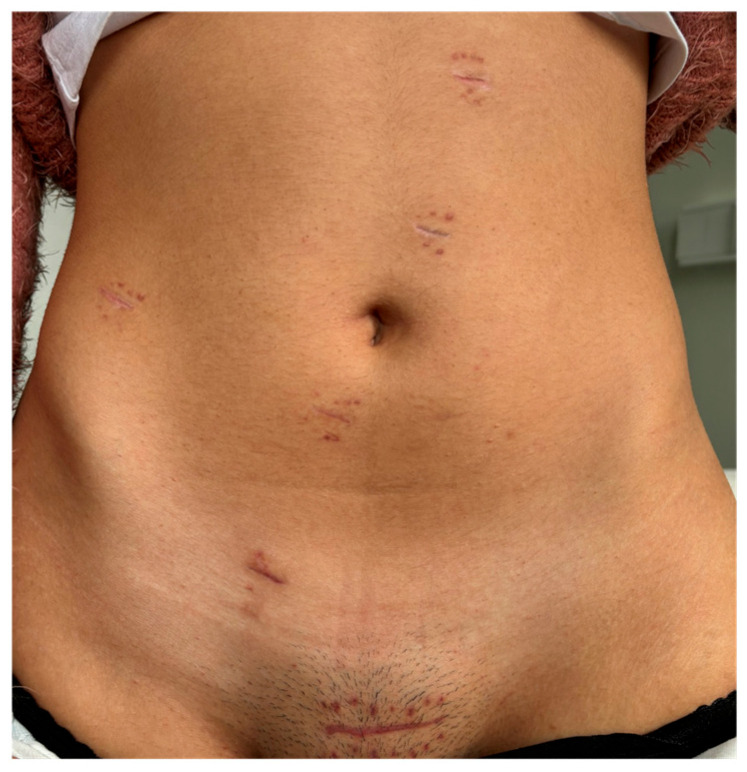
Abdomen aspect thirty days after surgery.

One month later, she had four bowel movements during the day and one at night. The patient had no complaints then, and her abdominal appearance was satisfactory (Figure 7).

## 4. Discussion

The da Vinci surgical system by Intuitive Surgical, Sunnyvale, CA, USA, is currently the gold standard in robotic-assisted surgery due to its surgeon-friendly interface and market penetration. The main issue with the da Vinci system is the costs associated with the initial investment and the final price of the procedure. This high cost is a significant obstacle to its worldwide implementation. After two decades of da Vinci’s monopoly, new robotic platforms have been approved for clinical use. One such system is the Hugo RAS**^®^**, which is as safe as the da Vinci system and offers reduced costs. This platform was recently introduced to the European market. The main surgical experience with this platform is in urology, with some international publications [8,9,10]. In Europe, experience with Hugo RAS**^®^** in colorectal surgery is limited [11,12,13,14,15], especially regarding total colectomy. This is the first published case of robotic-assisted total colectomy using the Hugo RAS**^®^** platform worldwide. The necessity of working in more than one abdominal quadrant in colorectal surgery implies greater complexity and added difficulty in robotic technical planning [16].

Santo António ULS, a university hospital in Porto, Portugal, acquired the Hugo platform in January 2023. The urology and general surgery teams, after a training program at the Orsi Academy with Medtronic support, began performing prostate, colorectal, and obesity surgeries. The first surgeries were performed in April 2023. Since then, eighty colorectal surgeries have been performed. Recently, a total colectomy in a selected patient was performed successfully. Upon reviewing the literature, we found that little has been written about the experience in robotic total colectomy, particularly with the Hugo system. Compared with the da Vinci Xi system, the apparent advantage of robotic-assisted total colectomy with Hugo is the reduction of costs, which can influence the decision to implement one system over the other.

For this reason, we describe our first experience with the Hugo RAS system, focusing on the technical aspects of the procedure and the challenges of managing this platform. Additionally, we reflect on the pros and cons of robotic versus laparoscopic total colectomy and the importance of selecting the appropriate patient for a robotic approach in centers with limited experience like ours.

There are several differences between the da Vinci and Hugo platforms. The main difference is the existence of four independent arms. The positioning of the arms is crucial for the ease and feasibility of the procedure to avoid arm collision and to enable use in different quadrants. The challenge is more significant in total colectomy because the port locations must be chosen for the right colectomy (Figure 2) and then adjusted for the left colectomy (Figure 4). The number and position of the ports differ from how we handle them in the Hugo RAS system compared with how high-volume centers do with the da Vinci Xi system [4,5]. Additionally, during the operation, it is necessary to change the surgical table inclination, redock the arms, and change the position of the instruments in the arms. This step may take more time with the da Vinci Xi. Other technical differences include the use of an assistant port (Figure 2 and Figure 4) for introducing devices for insufflation, smoke evacuation and filtration, and laparoscopic instruments when necessary, such as shears, Hem-o-lok endoscopic applicator, aspirator device, and endoscopic stapler (Signia™). To avoid creating an additional port for the assistant during the right colectomy, port 4 serves as the assistant port, alternating with the surgeon’s right hand (monopolar curved shears) (Figure 2). The colorectal transition section with a Signia™ stapler was introduced through the assistant port, and the sigmoid colon retracted with a grasper; the robotic phase ended. The assisted laparoscopic phase was initiated and performed as described. The Hugo platform has not yet approved/certified robotic staplers and vessel sealer devices. Currently, Hugo RAS energy devices are limited to bipolar and monopolar. We prefer to use the Hem-o-lok system for vessels of larger caliber and the Signia™ laparoscopic stapler for anastomoses. This approach helps us overcome Hugo’s current limitations compared with da Vinci.

Despite these complex technical aspects, we safely performed the assisted total colectomy, achieving an operative time, hospital stay, and recovery similar to or better than laparoscopic total colectomy performed on other patients with the same BMI, age range, and underlying condition.

This success can be achieved only in selected patients. Generally, laparoscopic surgery remains the gold standard for centers with limited experience in robotic-assisted colorectal surgery—offering shorter operative times, lower costs, and similar or better short-term outcomes regarding hospital stay and patient recovery.

## 5. Conclusions

The Hugo RAS platform is one of the emerging minimally invasive robotic systems that can be used for total colectomy with proper patient selection. The placement and choice of the arms and trocars were crucial to obtaining a similar operative time to the standard laparoscopic approach or the da Vinci Xi boom-mounted system.

The certification of Hugo’s new instruments, such as energy devices and staplers, will make this platform even more competitive. Parte superior do formulário. Parte inferior do formulário.

## Figures and Tables

**Table 1:** Main technical aspects of the procedure.

Phase	Docking Time	Patient Position	Port Placement and System Configuration	Main Steps
**Phase I** **Initial Steps and System Configuration**	**Docking Time:** 3 min 25 s	- Operating table at 60 cm height; - Supine position in modified lithotomy; - 10° Trendelenburg; - 10° left lateral tilt.	**Ports:** - Four ports positioned diagonally from the left hypochondrium to the right iliac fossa; - Two robotic trocars of 8 mm; - Two robotic trocars of 11 mm (one for the camera, one for traditional laparoscopic instruments); - A 12 mm trocar for insufflation and assistance. **System Configuration:** - Arm 1 (8 mm): Tilt (+30°), dock, 45°—reserve; - Arm 2 (11 mm): Tilt (+30°), dock, 145°—endoscope/assistant port; - Arm 3 (8 mm): Tilt (−15°), dock, 225°—surgeon’s left hand (bipolar); - Arm 4 (11 mm): Tilt (−15°), dock, 315°—surgeon’s right hand (monopolar curved shears).	- Port placement; - System configuration and robotic arm docking.
**Phase II** **Assisted Robotic Right Colectomy**	N/A	Same as Phase I	As configured in Phase I	- Access to retroperitoneum between the superior mesenteric vein and ileocolic vessels; - Dissection and division of ileocolic vessels; - Identification and control of right colic and middle colic vessels; - Medial-to-lateral mobilization of the right colon; - Opening of the greater omentum to access the lesser sac and complete mobilization of the right colon.
**Phase III** **System modification**	**Docking Time:** 2 min 55 s	- 10–15° Trendelenburg; - 10–15° right lateral tilt.	**System Configuration:** - Arm 1 (8 mm): Tilt (−15°), dock, 45°—surgeon’s left hand (Cadiere grasper); - Arm 2 (8 mm): Tilt (−15°), dock, 145°—surgeon’s right hand (monopolar curved shears); - Arm 3 (11 mm): Tilt (+30°), dock, 225°—endoscope; - Arm 4 (11 mm): Tilt (+30°), dock, 315°—reserve (Cadiere bipolar forceps).	- Detachment of robotic arms; - Patient repositioning; - Redocking of robotic arms with a new configuration for left colectomy.
**Phase IV** **Assisted Robotic Left Colectomy**	N/A	Same as Phase III	As configured in Phase III	- Division of inferior mesenteric artery and vein; - Medial-to-lateral dissection of the left mesocolon; - Opening of the left paracolic gutter up to the splenic flexure; - Mobilization of the splenic flexure; - Disconnection of the greater omentum from the left transverse colon; - Complete mobilization of the transverse colon; - Partial mesorectal dissection and division of the rectum below the rectosigmoid junction.
**Phase V** **Laparoscopic Assisted Phase**	N/A	- Suprapubic (Pfannenstiel) incision	- Removal of robotic trocars; - Use of traditional laparoscopic instruments.	- Incision for extraction of surgical specimen; - Division of the ileum and introduction of a 28 mm EEA circular stapler anvil; - Transanal ileorectal anastomosis; - Integrity test of the anastomosis with methylene blue; - Closure of the ports.

## Data Availability

The original contributions presented in the study are included in the article, further inquiries can be directed to the corresponding author.
